# Perceptions of Care Sport Connectors’ Tasks for Strengthening the Connection Between Primary Care, Sports and Physical Activity: A Delphi Study

**DOI:** 10.5334/ijic.4789

**Published:** 2020-04-01

**Authors:** Eva Smit, Karlijn E. F. Leenaars, Annemarie Wagemakers, Koos van der Velden, Gerard R. M. Molleman

**Affiliations:** 1 Academic Collaborative Centre AMPHI, Primary and Community Care, Radboudumc, Nijmegen, NL; 2 National Institute for Public Health and the Environment, Bilthoven, NL; 3 Health and Society Group, Department of Social Sciences, Wageningen University & Research, Wageningen, NL

**Keywords:** physical activity promotion, intersectoral collaboration, Delphi method, care sport connector

## Abstract

**Introduction:**

Care sport connectors stimulate physical activity and facilitate collaboration between the primary care and physical activity sectors in the Netherlands. To strengthen intersectoral collaboration between the primary care and sports sectors, it is necessary to study which tasks a care sport connector must fulfil according to their own and other professionals’ perceptions.

**Methods:**

A Delphi study was conducted with 182 professionals from the primary care, public health and physical activity sectors. Rounds 1 and 2 included questions about task perception, willingness to collaborate and expectations of care sport connectors. Rounds 3 and 4 were used to reach consensus.

**Results:**

All professions acknowledged physical activity promotion tasks, but they are not all willing to collaborate. They expect a broad range of roles from care sport connectors: informative, executive, guiding and intermediate. Care sport connectors reached consensus on two roles: informative and intermediate.

**Discussion:**

Care sport connectors have an important role in strengthening intersectoral collaboration. All the professions acknowledged a task concerning physical activity promotion and accepted a broker role. Thus, a public health mind-set seems to be present to some extent. However, challenges remain, such as the lack of willingness to collaborate among primary care professionals and sports policies not (yet) supporting intersectoral collaboration.

## Introduction

Physical activity is an important determinant of health and is beneficial for the prevention of chronic diseases [1]. Therefore, in 2012, the Dutch Ministry of Health, Welfare and Sport began appointing neighbourhood sports coaches (*buurtsportcoaches*), to whom a broker role is ascribed [2]. Neighbourhood sports coaches facilitate a community-level connection between the physical activity sector and other sectors with the aim of stimulating inactive people to become physically active. Some neighbourhood sports coaches especially focus on collaboration between the primary care and physical activity sectors: they are called care sport connectors. The general idea is that care sport connectors facilitate the connection between the primary care and physical activity sectors. Professionals from these sectors then collaborate and implement lifestyle interventions, which reach certain target groups. Eventually, these target groups will become able to self-manage their physical activity: they will become more physically active in their neighbourhoods and their health outcomes will improve. However, there is no blueprint for this function and the implementation of it. In other countries, there are also professionals within the care domain that encourage exercise or in the sports and exercise sector that pay attention to health aspects. But a separate professional with a broker function such as the care sport connector is typically Dutch, as far as we know. At the same time, intersectoral collaboration is challenging. For example, there is no history of different sectors working together, roles and responsibilities are not clear and there are differences in knowledge and communication skills [3].

A broker role seems promising [4] because it is challenging to increase physical activity and to consequently influence health. This is due to interrelated determinants that contribute to lifestyle behaviours at multiple levels: individual, social, environmental and policy [5]. An integrated approach is required to link the talents, resources, relationships and approaches of different sectors and professions, and to affect all these interrelated determinants more effectively, efficiently and sustainably than one sector or profession could achieve alone [3,5,6,7]. Therefore, an integrated approach requires intersectoral collaboration which means working together to combine talents and strengths to achieve a common goal [8]. As the care sport connector is new as well its area of inquiry and action, conceptual clarification is demanded [9]. The Healthy Alliances (HALL) framework seems to be a useful framework to study the factors that hinder or facilitate the success of intersectoral collaboration and has been successfully used to study intersectoral collaboration initiated by care sport connectors [10] and by youth-care organisations [11], amongst others. The HALL framework identifies three clusters of factors. At the institutional level factors are policy, planning horizons and funding. At the (inter)personal level factors are attitudes and beliefs, self-efficacy, social identity and relationships. At the organisational level factors are a shared mission, visibility, roles and responsibilities, flexible time frames, building on capacities and a communication structure.

A systematic literature review [12] revealed barriers to intersectoral collaboration between the primary care and physical activity sectors. These include different shared interests, different cultures, a lack of communication, unclear roles and responsibilities, a lack of time and a lack of knowledge. This might explain why intersectoral collaboration is hard to establish [3,8], and why transfer rates of patients from the primary care sector to the physical activity sector are low. As such, a broker role might be promising for overcoming these barriers [4,10].

Previous interviews with care sport connectors revealed that they can make contact with primary care and sports professionals [10]. They establish networks with general practitioners, nurse practitioners, physiotherapists, employees from municipal health services, social neighbourhood teams and sports or physical activities clubs. However, structural intersectoral collaboration is hard to reach and, in this phase, collaboration mainly occurs on a project basis [10]. In their broker role for intersectoral collaboration, care sport connectors depend on the resources, approaches and relationships between and with other community professionals. Since there is no blueprint for the new care sport connector function, it is not fully clear which role a care sport connector can fulfil in the system for physical activity promotion according to other concerned professions.

Our previous studies [10,13] showed the perceptions of a limited number of care sport connectors and professionals from their experience within the network of a particular care sport connector. However, it is also relevant to reveal the overall perceptions of professionals towards the care sport connector function, their task execution and their willingness to collaborate intersectorally in a more general and comprehensive way. This knowledge will show the potential added value of the care sport connector function and can yield a direction for the role and task profile of care sport connectors in the system of physical activity promotion.

Therefore, the main question of this exploratory study is: ‘Which tasks must a care sport connector perform according to their own and other professionals’ perceptions to strengthen the connections between primary care, sports and physical activity in order to stimulate physical activity?’ To answer this question, we require insight into the following sub-questions:

1. Which tasks do primary care and sports professionals see for themselves to stimulate residents to be physically active?
2. How and on what kind of basis would these professionals like to collaborate with other professionals in the community to stimulate residents to become physically active?
3. Which tasks do other professionals expect from the care sport connector and are these expectations in line with the task perception of care sport connectors?

## Method

### Delphi method

**Figure 1:**
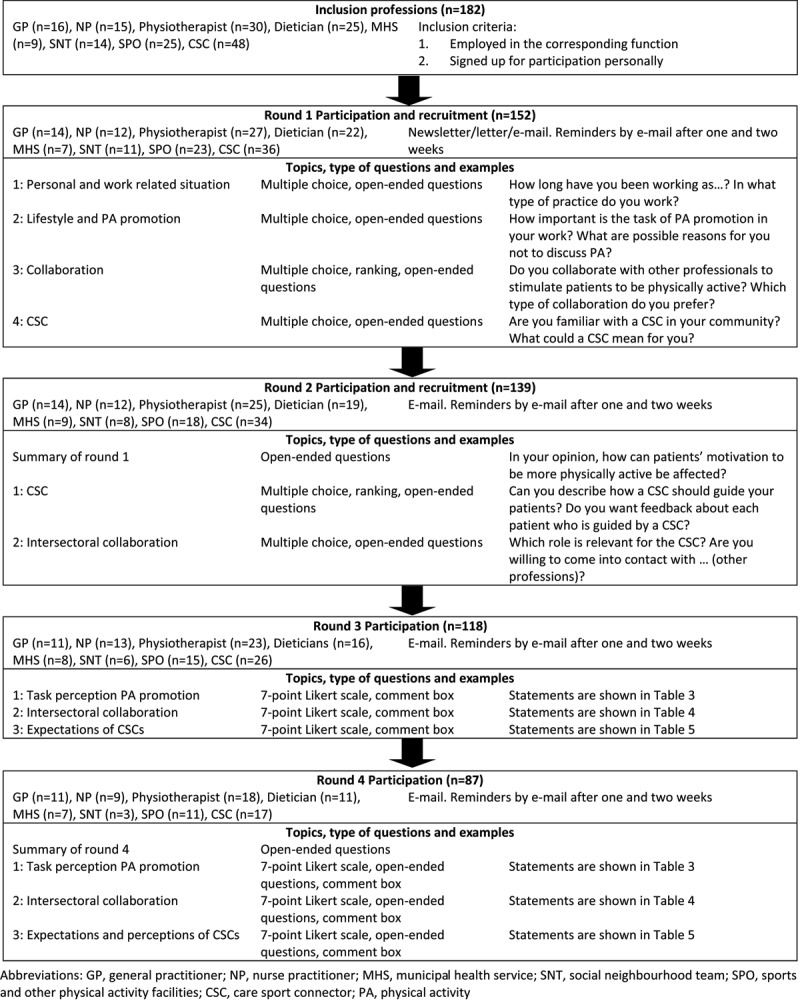
Overview of the study procedure.

In 2015, a Delphi method was used to obtain a full view of opinions and interests of diverse professionals [14,15] and to reliably reach consensus between a group of professionals in subsequent rounds [16,17]. Feedback about previous rounds was controlled by the researcher and gives participants the opportunity to score statements differently than they did earlier [18]. Participants remain anonymous [18] and are not allowed to participate in the third and fourth rounds if they did not participate in the first or second rounds (Figure 1).

### Participants

A purposive sampling strategy was used to invite professionals who work in the community, are employed in the primary care or sports sectors, and are potential contacts of the care sport connectors, based on previous interviews with care sport connectors [10]. As a result, we included general practitioners, nurse practitioners, physiotherapists, dieticians, municipal health services, social neighbourhood teams, sports clubs and other physical activity facilities and care sport connectors. Potential participants were approached in four ways:

1. Care sport connectors, physiotherapists and sports club and other physical activity facilities were approached via the newsletters of our consortium partners (i.e., the Association of Sports and Municipalities (VSG), Royal Dutch Society for Physical Therapy (KNGF), Knowledge Centre Sports & Physical Activity (KCS) and, Dutch Olympic Committee * Dutch Sport Federation (NOC*NSF)), which are representatives of these professions.
2. 300 general practitioners, nurse practitioners and dieticians were randomly selected from the Radboudumc database, which includes professionals from all over the country, and invited with a personal letter. Due to a low response rate by general practitioners (3%), an additional group of seven general practitioners was assigned in response to an invitation based on the address list (n = 55) of the Radboudumc department of Primary and Community Care, which includes general practitioners mainly situated in the area of Radboudumc.
3. All 25 municipal health services in the Netherlands were invited by e-mail to delegate one employee from the department of health promotion.
4. We invited 90 social neighbourhood teams by e-mail according to available data on the internet.

Professionals could sign up for the study; if they met the inclusion criteria (i.e., still in a corresponding function), they were included. We hoped to include 10–15 professionals from each selected profession, because homogenous panels with 10–15 professionals would lead to sufficient results [17].

The response rates were 83.5% (n = 182) for round one, 76.4% (n = 182) for round two, 73.01% (n = 167) for round three and 52.1% (n = 167) for round four. Reasons for non-response were time constraints, vacation, sick leave, maternity leave, job change and lack of knowledge. Fifteen participants did not participate in round 1 or 2 and were not invited for the subsequent rounds. Information about each profession is described in Table 1.

**Table 1:** Characteristics of the participating professionals.

Profession	Age (years)	Gender	Work experience (years)	Type of practice	Population (%)

GP (16)	44.79 (33–62)	7 men 9 women	15.43 (2–34)	3 solo practice 3 duo practice 4 group practice 2 primary care centre 3 health care centre 1 observer	Seniors: 31.07 (4–70) Natives: 74.43 (1–99) Low education: 44.29 (10–85) Single parent families: 17.43 (5–41)
NP (15)	45.17 (27–56)	1 men 14 women	8.63 (2–15)	2 solo practice 3 duo practice 4 group practice 6 health care centre	Seniors: 40.08 (9–80) Natives: 51.55 (2–95) Low education: 38.55 (8–65) Single parent families: 19.36 (0–40)
Physiotherapists (30)	44.29 (26–59)	12 men 18 women	21.98 (2–38)	2 solo practice 1 duo practice 16 group practice 7 health care centre 2 nursing homes 2 different	Seniors: 54.36 (1–100) Natives: 61.12 (4–99) Low education: 39.19 (9–81) Single parent families: 26.12 (7–75)
Dieticians (25)	40.35 (21–59)	25 women	17.54 (4–40)	9 solo practice 6 health care centre 2 nursing homes 2 hospitals 1 rehabilitation centre 2 home care 3 different	Seniors: 49.50 (5–96) Natives: 47.22 (4–96) Lower education: 43.22 (5–100) Single parent families: 17.23 (2–50)
MHS (9)	35.29	2 men 7 women	5.18		
SNT (14)	43.78	6 men 8 women	1.72		
SPO (25)	42.95	17 men 8 women	5.4	1 walking group 3 fitness 3 (table) tennis 5 football 1 korfball 5 field hockey 1 gymnastics 1 swimming 5 multiple sports	
CSC (48)	33.06 (22–57)	23 men 25 women	3.08 (0.16–15)		Seniors: 33.3 Youth: 30.55 Adolescents: 19.44 Adults: 8.33 Inactive people (all ages): 2.78 Disabled/with a chronic condition: 25 All ages: 25

Abbreviations: GP, general practitioner; NP, nurse practitioner; MHS, municipal health service; SNT, social neighbourhood team; SPO, sports and other physical activity facilities; CSC, care sport connector; PA, physical activity.

### Procedures

Before beginning the Delphi study, we set guidelines for each round. Each round would take no more than 30 minutes for the respondents, who would get two weeks to fill in the questionnaire. They received reminders after one week and two weeks. We would analyse results in two weeks and send subsequent questionnaires one month after the previous one [18].

To avoid an excessive load for the respondents, two rounds with open and closed questions were used to collect a range of views and issues about predetermined topics. The questions in the first two rounds were operationalised based on the factors of the HALL framework [8] and refined in an expert panel (n ≥ 3) for each profession. In this manner, questionnaires were adapted in line with the daily practice of each profession and became profession-specific. The first round consisted of questions about the professionals’ personal and work-related situations, lifestyle and physical activity promotion (relevance, extent, barriers and facilitators), inter-professional and intersectoral collaboration (manner, partners, barriers and facilitators) and care sport connectors (contact, usefulness). The second questionnaire addressed care sport connectors (referral scheme, expectations) and intersectoral collaboration (expectations, contribution, based on a review) [12]. The professionals were also given a summary of the previous round to give them insight into the answers from all the professions.

The third round consisted of statements based on the qualitative analysis of the answers on open-ended questions from the first two rounds. Since answers differed by profession, the statements were formulated differently for each profession. This created a Delphi study for each profession without methodical cross-pollination. Statements were divided by topic: task perception of physical activity promotion, intersectoral collaboration and expectations of care sport connectors. Statements were scored on a 7-point Likert scale from totally disagree (1) to totally agree (7). After each topic, participants could add information in a comment box.

In the fourth round, statements without consensus were resubmitted to each profession. At the beginning of each topic, we gave an overview of the previous round to show the statements for which consensus was reached. After each overview, professionals were asked if that consensus was appropriate; if not, they were asked to indicate what was missing or inappropriate. If a professional did not agree with a statement (did not choose the last two categories), they were asked why.

### Analysis

The qualitative data from the first two rounds was analysed according to the six steps of Creswell [19]. In the first step, the data were organized and prepared for analysis. In the second step, the transcripts were read. In the third step, the transcripts were coded and analyzed using software for qualitative analysis (Atlas.ti, version 7.1.5). In the fourth step, the codes were clustered into themes, where in steps 5 and 6 more bottom-up codes were assigned to the various themes. The emerging themes were input to formulate statements for rounds three and four [15,18]. Two researchers (ES and MH) analysed data and formulated statements; they discussed differences in interpretation to reach consensus among the researchers. Answers, comments and statements from each round were discussed with the research team to reach agreement about the content for rounds three and four. Comments from those rounds as provided by the respondents were classified in facilitators and barriers to gain insight into why a statement was rejected or accepted.

Quantitative data was analysed with SPSS version 22 to calculate the mean, median, interquartile range and percentage. Interquartile ranges were calculated to provide insight into the distribution of answers. According to Powell’s review [15] we decided beforehand [20] that consensus would be reached if 80% of the answers fell in the two outermost categories of the Likert scale [18]. For the fourth round, each third-round statement was classified in one of the following categories according to the corresponding decision rules: infeasible (mean < 5.5), indecisive (mean ≥ 5.5 and no consensus) or consensus (80% of the answers were in the outermost categories). Indecisive statements were included in the fourth-round questionnaire. Infeasible statements (n = 59 for all professions) were excluded to avoid response exhaustion, keeping in mind that convergence of opinion would happen in subsequent rounds, but be comparatively slight [21,22].

## Results

We present the results of each theme with most relevant statements; the full overview of statements can be found in the appendixes. Reasons to reject or accept a statement are shown in Table 2 with the facilitators and barriers for each theme as listed by respondents. These reasons are also highlighted by quotes to give a more comprehensively insight.

**Table 2:** Reasons for respondents to accept or reject a statement in rounds 3 and 4.

Profession	Facilitators	Barriers

**Physical activity promotion task**		
GP	Secondary prevention	Time pressure; No priority; No general advice; Activity costs; Impossible to get an overview of activities; Patient’s responsibility
NP		Patient’s physical state; Time pressure; Knowledge deficit; Unreachable to stay up to date about the activities
Physiotherapist	CSC guidance; Fittest; Patient’s pleasure/wish; Final stage of care	Concurrent activities; Impossible to get an overview of activities; Guidance by physiotherapist; No reimbursement
Dietician	CSC guidance; Current network;	Reaching the target group; Only if PA is relevant to discuss; Permission is needed; Update social cart; Knowledge deficit; Time pressure; Physiotherapist too expensive
SNT	Our task to succession; Using own strength; Own guidance; Guidance of CSC; Formulation of goals and actions PA as a means	High caseload; Patient needs guidance of SPO; Own responsibility
MHS	Other professionals; Local support hours	If necessary, not constant; Collective approach
SPO	Free sports are not motivating; Flexible membership	Traditional function is the primary task; Noncommittal; Hard to form a team; Own responsibility; Deficit of frame
**Collaboration**		
GP	Advise; Availability of nurse practitioner; Use of other means	Not our task; Priority; Time consuming; Patient’s responsibility
NP	Existing network; Motivated NP; Knowledge of each other’s existence is enough	Time consuming; No reimbursement; GP’s permission; Not our task
Physiotherapist	Essential element	No reimbursement
Dietician	Interplay of disciplines; Strengthens practice; Other means for information distribution	Concurrence; Time consuming; No reimbursement
SNT	Network is key	
MHS	Focus on the arrangement	Time consuming
SPO	Skilled CSC; Own contact	Time consuming; No volunteers
**Expectations Care Sport Connectors**		
GP		Knowledge deficit about CSCs
NP	Integrated care organization; Feedback	No contact with CSCs; Patient’s permission; Patient’s responsibility CSC should connect to my network; Unclear role of CSCs
Physiotherapist	Guidance CSC	CSC should connect to my network; Knowledge deficit about CSCs Concurrence; Unclear role of CSCs
Dietician		CSC should connect to my network; Patient’s permission; Knowledge about CSCs; Patient’s responsibility; Unclear role of CSCs
SNT	Guidance of CSC Give substance to goals and actions	Working hours of CSC; Unmotivated group; No CSC; A member of SPO should be the CSC; CSC should connect to my network
MHS	Guidance of CSC	Intermediary role; CSC’s role is context dependent
SPO		Role dependent on municipality; Unclear role of CSC
CSC	Collaboration Other coordinating parties Existing networks	Direction is intermediary instead of executive; No individual approach – other colleague; Difficult target group; Demand driven; Content PA only; Medical knowledge deficit; Municipality chooses direction

Abbreviations: GP, general practitioner; NP, nurse practitioner; MHS, municipal health service; SNT, social neighbourhood team; SPO, sports and other physical activity facilities; CSC, care sport connector; PA, physical activity.

### Professions’ tasks concerning PA promotion

All the professionals acknowledged tasks related to PA promotion (which were different for each profession) and declared that their profession’s task profile was realistic (Table 3 and Appendix A).

**Table 3:** Professions’ tasks concerning PA promotion.

Statements	GP	NP	PH	DI	SNT	MHS	SPO

**Goals:**							
As a professional, it is my task to pay attention to physical activity promotion in the daily lives of patients	C*	C	C	C	C	C	–
	1*	1	1	1	1	1	
**Inform:**							
As a professional, it is my task to provide patients insight into the necessity and importance of getting a sufficient amount of physical activity	C	C	C	C	–	C	–
	0	1	1		1	1	
As a professional, it is my task to provide patients insight into the possibilities for staying physically active in daily life	NC	NC	C	NC	C	NC	–
	1	1	1	2	2	1	
**Refer:**							
As a professional, it is my task to motivate patients to be physically active in their daily routine	C	C	C	C	–	–	–
	0	1	1	1			
As a professional, it is my task to try to be, as much as possible, aware of the regular sports and physical activities that are present in the neighbourhood	NC	NC	C	C	C	NC	–
	2	0	1	1	1	2	
As a professional, I will actively refer patients to regular sports and physical activities in the neighbourhood if these are suitable for the patient	NC	NC	C	C	–	–	–
	2	0	1	2			
**Execute:**							
As a professional, it is my task to use physical activity as a means	–	–	–	–	NC	NC	–
					4	1	
If a CSC asks me to, I am willing to give group sessions to inform people about the need and benefits of sufficient physical activity	NC	NC	C	–	–	–	–
	4	3	1				
If a CSC asks me to, I am willing to offer sports and physical activities for people with (an increased risk for) health problems	–	–	–	–	–	–	NC
							1
If a CSC asks me to, I am willing to be a social involved club	–	–	–	–	–	–	C
							1

C, consensus; C*, consensus reached in 4th round due to a lower response rate; NC, no consensus reached; –, statement was not provided to this profession. Interquartile range is presented for each statement, with a occurred range from 0–4. Abbreviations: GP, general practitioner; NP, nurse practitioner; PH, physiotherapist; DI, dietician; SNT, social neighbourhood team; MHS, municipal health service; SPO, sports and other physical activity facilities; CSC, care sport connector.

General practitioners mainly reached consensus about tasks intended to inform patients about physical activity. Referral tasks were limited to referring patients to a physiotherapist and motivating patients to become physically active. They mentioned the barrier of time pressure and indicated the need to prioritize. Physical activity was promoted if it was relevant to the patient’s complaint. Consensus was not reached about the tasks of being aware of the regularly available sports and physical activities in the neighbourhood or referring patients to these activities. General practitioners indicated that it is impossible for them to be informed about the constantly changing activities and the quality of trainers for these activities, which they view as necessary to give appropriate advice to patients.

“Whether lifestyle and physical activity will be discussed comprehensively or concisely depends on the time constraints during a consult. Informing patients does not take much time, but motivating them does. It could always be addressed.” (General practitioner, 15)

However, nurse practitioners, whose tasks are delegated by general practitioners, mentioned that it is their task to talk with patients about their individual possibilities. They use the social cart with physical activities to offer possibilities for staying physically active. But they also declared that there is not enough time to be aware of all the activities in a neighbourhood and that they do not have enough knowledge to refer patients to a specific activity.

“I find it is also a task of a nurse practitioner to motivate patients to become physically active and to discuss individual possibilities.” (Nurse practitioner, 20)

Physiotherapists, on the other hand, reached consensus for each task that was presented to them and acknowledged having a role in informing patients about the necessity and benefits of physical activity, referring patients and executing tasks concerning physical activity promotion. In their view, most of these tasks are relevant in the final stage of a patient’s treatment.

Dieticians reached consensus about tasks concerning physical activity promotion (inform and refer) even though their core business is focused on nutrition. They stated that their profession focuses on health promotion, whereby physical activity and nutrition are intertwined. However, there is not always enough time to focus on physical activity promotion if the nutritional problem is too complicated. Another barrier they described is that they believe that they are not a physical activity specialist and did not learn enough about it in their studies to give insight into or advise about suitable physical activities.

Social neighbourhood teams play a role in prevention, and the promotion of physical activity is part of this task. They stimulate residents to be physically active and acknowledge that they must be aware of the arrangement of physical activities in the neighbourhood and use the social cart to give residents insight into possibilities to become physically active. Physical activity is not only a goal, but is also used as a mean.

“Physical activity and health are an important part of total self-reliance.” (Social neighbourhood team, 169)

The health promotion departments at municipal health services have the goal of promoting health. This task is executed collectively, which explains why municipal health services did not reach consensus about tasks on an individual level. Municipal health services acknowledged the tasks of increasing residents’ knowledge of physical activity and detecting their need to become physically active. Some departments reported having local support hours that enable them to act on an individual and/or neighbourhood level.

Trainers and supervisors of sports and other physical activity facilities recognized their task in the execution of physical activities for people with (or at an increased risk for) health problems. However, they pointed out that it is challenging to perform these new tasks in addition to their traditional function. They mentioned lacking a framework, such as professional trainers, knowledge, finances and sufficient participants, for new activities.

“Our counsellors are volunteers; you can’t expect a club to be able to find volunteers who are familiar with multiple disabilities.” (Sports clubs and other physical activity facilities, 168)

### Willingness to collaborate

Willingness to collaborate with other professionals to stimulate physical activity showed a varied picture. While a few professions (general practitioners and nurse practitioners) did not reach consensus for the statements about collaboration, other professions (physiotherapists and social neighbourhood teams) did reach consensus for all the statements about collaboration (Table 4 and Appendix B).

**Table 4:** Intersectoral collaboration.

Statements	GP	NP	PH	DI	SNT	MHS	SPO

**Goal:**							
As a professional, I am willing to collaborate with other professionals in the neighbourhood to stimulate residents to be physically active	NC	NC	C	C	C	C	C
	2	1	1	1	1	1	1
**Participate in meetings:**							
As a professional, I will participate in (network) meetings in the neighbourhood that are dedicated to promoting a healthy lifestyle among neighbourhood residents	NC	NC	C	C*	C	C	C*
	3	2	1	1	1	1	3
**Arranging activities:**							
As a professional, I am willing to develop multidisciplinary programs with other professionals to promote the overall lifestyles of participants	NC	NC	C	NC	C	C	NC
	3	2	1	2	2	1	1
As a professional, I am willing to contribute to activities organized in the district to promote a healthy lifestyle (e.g., fitness tests, health fairs)	NC	NC	C	NC	C	–	C
	3	3	1	1	2		1
**Contact:**							
As a professional, I would like to become acquainted with sports and exercise professionals or health and welfare professionals from the neighbourhood	NC	NC	C	C	C	NC	C*
	1	1	1	1	1	3	1
As a professional, I would like to have contact with sports and exercise groups from the neighbourhood	NC	NC	C	NC	C	NC	NC
	2	2	1	2	1	2	2

C, consensus; C*, consensus reached in 4th round due to a lower response rate; NC, no consensus reached; –, statement was not provided to this profession. Interquartile range is presented for each statement, with a occurred range from 0–4. Abbreviations: GP, general practitioner; NP, nurse practitioner; PH, physiotherapist; DI, dietician; SNT, social neighbourhood team; MHS, municipal health service; SPO, sports and other physical activity facilities.

General practitioners and nurse practitioners explained that they do not recognize statements about collaboration to be their task and worried that it would take a lot of time at the expense of primary patient care. The situation and extent of collaboration could change this view: they mentioned being informed by other means (e.g., mail or telephone).

“All consultations and/or the additional deployment of an nurse practitioner is not funded by the health insurer. This means that it is entirely dependent on the motivation of the NP and whether the employer (General practitioner) agrees with the hours which will be devoted to this.” (Nurse practitioner, 28)

Physiotherapists and social neighbourhood teams reached consensus for each presented statement regarding participation in meetings, arranging activities and contact. Physiotherapists stated that collaboration is essential to promote physical activity because if a professional is informed about other professionals’ qualities and abilities, they can make a concrete referral or advice. According to physiotherapists, consultation is necessary for the implementation and progress of projects considering physical activity promotion. This is in line with social neighbourhood teams who stated that, with a proper network in the community, it is possible to find people who need to be motivated to become physically active and to accommodate them in a suitable activity.

“Good relationships with physical activity providers are essential for a concrete referral to or a concrete advice about a physical activity. Otherwise, patients have to change their lifestyle on their own and become physically active. This will be a large barrier for a large group of patients and consequently their potential intention to change will be delayed or ultimately not realized.” (Physiotherapist, 41)

Dieticians agreed on collaborating with other professionals to promote physical activity. They indicated that they would like to execute the tasks of physical activity promotion as an interplay with other professionals and declared that it would strengthen their own practice. However, collaboration and meetings require a time investment and consequently cost money. Therefore, dieticians affirmed that collaboration should be profitable or declarable and stated that they preferred other ways to collaborate or share information, if possible.

“What applies to all networking events: it takes a lot of time and, probably, a lot of information could be given on paper or somehow similarly. I am not opposed to network meetings, but goals and methods should be clearly described. And they should yield profits for my own participation.” (Dietician, 72)

These time constraints were also mentioned by municipal health services and sports club and other physical activity facilities, who both reached consensus about several statements regarding collaboration.

“Keeping in touch is very time consuming. Preferably you do that as a club, but that is almost impossible if the association is managed by volunteers.” (Sports clubs and other physical activity facilities, 151)

Municipal health services stated that they focus on forming and strengthening networks and programs instead of knowing each professional in each community.

### Task expectations and perceptions

All the professions, except nurse practitioners, would accept a care sport connector contacting them about physical activity promotion (Table 5 and Appendix C).

**Table 5:** Expectations and perceptions of Care Sport Connectors’ tasks.

Statements	GP	NP	PH	DI	SNT	MHS	SPO	CSC

**Goals:**								
As a professional, I see the work of a CSC as an addition to my work	–	NC	C	–	–	–	–	–
		1	1					
As a professional, I am open to a CSC contacting me	C	NC	C	C	C	C	C	–
	0	1	1	1	1	0	1	
**Informative:**								
As a professional, I expect a CSC to be aware of the sports and physical activities in the neighbourhood	C	C	C	C	C	C	C	C
	1	1	1	1	1	0	1	1
As a professional, I expect a CSC to map the sports and physical activities	C	C	C	C	C	C	C*	C
	1	1	1	1	1	1	2	1
As a professional, I expect a CSC to keep me informed about current sports and physical activities	NC	C	C	C	C	NC	–	C
	1	1	1	2	1	3		1
As a professional, I expect a CSC to create awareness of his/her function and its professional potential for us	C	C	C	C	C	–	–	C
	1	1	1	1	1			1
As a professional, I expect a CSC to transfer knowledge which is necessary for the provision of sports and physical activities for people with (an increased risk for) health problems	–	–	–	–	–	–	NC	C
							1	1
**Executive:**								–
As a professional, I expect a CSC to arrange easily accessible sports and physical activities	C	C	C	C	–	C	–	C*
	1	0	1	2		1		1
As a professional, I expect a CSC to arrange sports and physical activities that meet the wishes and needs of the target group	–	–	–	–	C	–	–	C
					2			1
As a professional, I expect a CSC to provide support to recruit members with (an increased risk for) health problems	–	–	–	–	–	–	C	NC
							0	3
**Referral:**								
As a professional, I would use the guiding service of a CSC	C	C	C	C	C	–	–	–
	0	1	1	1	1			
As a professional, I expect a CSC to guide people, when necessary, to suitable sports and physical activities	–	–	–	–	–	C	–	NC
						1		1
As a professional, I expect a CSC to develop a buddy system, so people can exercise together instead of individually	C	C	NC	C	C	–	–	NC
	1	1	0	2	2			2
As a professional, I expect a CSC to monitor people to ensure they have made structural changes in behaviour	NC	NC	C	NC	–	–	–	NC
	2	2	1	1				3
As a professional, I expect a CSC to refer people back to me if they have physical complaints	C	NC	C	–	–	–	–	C
	0	1	1					1
As a professional, I expect a CSC to refer people back to me if they quit or do not show up at sports or physical activities	NC	NC	C	–	–	–	–	NC
	4	3	1					3
**Broker role:**								
As a professional, I expect a CSC to take a coordinating role concerning (network) meetings in the municipality	C	C	C	C	NC	NC	C*	NC
	0	0	1	1	2	1	1	1
As a professional, I expect a CSC to take a coordinating role to connect care, welfare and sports professionals in the neighbourhood	–	–	–	–	–	C	–	C
						1		1
As a professional, I prefer the CSC to act as an intermediary for the contact with sports and PA facilities or care professionals instead of maintaining contact with these professionals myself	C	NC	C*	C	NC	C	NC	C
	1	0	0	1	4	1	2	1
If a CSC asks me to, I am willing to help the CSC draft a plan of action for a physical activity intervention	–	–	–	–	–	C	–	–
						0		
If a CSC asks me to, I am willing to introduce a CSC to our network of healthcare professionals	–	–	–	–	–	C	–	–
						1		

C, consensus; C*, consensus reached in 4th round due to a lower response rate; NC, no consensus reached; –, statement was not provided to this profession. Interquartile range is presented for each statement, with a occurred range from 0–4. Abbreviations: GP, general practitioner; NP, nurse practitioner; PH, physiotherapist; DI, dietician; SNT, social neighbourhood team; MHS, municipal health service; SPO, sports and other physical activity facilities; CSC, care sport connector.

Professions expected that care sport connectors would publicize their function, allowing them to know what they could expect from a care sport connector, because this is still unclear.

“How can you have expectations if there is no clear guideline about what a care sport connector can and should do?” (Physiotherapist, 49)

Despite this lack of clarity, all the professions reached consensus about care sport connectors’ informative and executive roles concerning physical activities. They expect care sport connectors to be aware of sports and physical activities in the neighbourhood and to map these activities. Nurse practitioners, physiotherapists, dieticians and social neighbourhood teams also presume that a care sport connector would inform them about these activities. In addition, professionals expect care sport connectors to arrange or embed easily accessible physical activities.

Furthermore, all the professions agreed that they would use or expect the service of a care sport connector in guiding people to local sports or physical activities as a referral function. This guidance should be matched with individual needs and wishes and motivate people to become physically active. In line with these expectations, general practitioners, nurse practitioners, dieticians, and social neighbourhood teams expect care sport connectors to develop a buddy system so people can exercise together instead of individually.

“We lead the resident to the care sport connector, and the care sport connector should ensure a warm welcome. And he shouldn’t let the resident slip away.” (Social neighbourhood team, 177)

Additionally, care sport connectors could fulfil a broker role in facilitating collaboration. Expectations about these tasks varied. General practitioners, physiotherapists, dieticians and municipal health services prefer the care sport connector to be a broker between the primary care and sports sectors. In contrast, nurse practitioners, social neighbourhood teams and sports clubs and other physical activity facilities did not reach consensus about this statement and commented that it is better to maintain these contacts yourself.

“It is also good to stay in contact yourself, but this is not always possible (in the case of volunteers). Then it is fine if a care sport connector performs that role.” (Sports clubs and other physical activity facilities, 151)

This divided picture was also visible concerning the task for care sport connectors as coordinators of network meetings. Where primary care professionals demand this task from care sport connectors, social neighbourhood teams and municipal health services did not reach consensus. In addition, the municipal health services emphasized in the first two rounds that they could provide support for care sport connectors: they can help care sport connectors with writing project applications and action plans and, building and sustaining their networks and contributing to knowledge about the municipality where they work.

“Their main task is to guide vulnerable residents. The task of public health services and regional support structures is to bring networks together and organize knowledge meetings. Some care sport connectors like to perform this coordinating role, and this is possible if it fits the profile.” (Municipality health service, 32)

Care sport connectors rated the same statements but reached consensus on different statements regarding their tasks. Care sport connectors reached consensus about their informative tasks related to physical activities. They acknowledged that they need to be aware of the sports and physical activities in the community and that they should map these activities. They viewed it as their task to inform professionals about sports and physical activity offerings, developments regarding sports and physical activity, and the possibilities for referring people from primary care to the sports sector. Consensus about their executive function was reached due to a lower response rate. Many care sport connectors mentioned that they only arrange new activities if this is necessary and demand driven. Care sport connectors did not reach consensus about their referral function for guiding people to suitable sports and physical activities and setting up a buddy system. They declared that they will not perform their tasks on an individual level. On the other hand, they also mentioned that they would consider several possibilities if necessary to meet this demand. In several municipalities, a colleague is responsible for this task.

“I would not guide this person myself, but I would look for a volunteer or an institution who could perform this task.” (Care sport connector, 138)

Care sport connectors reached consensus about their broker role and the task of establishing new connections between the first and zero lines in the community and between the primary care and sports sectors. However, they did not reach consensus about taking a coordinating role in (network) meetings and organizing meetings. Care sport connectors mentioned that it is not necessary to coordinate or organize meetings if this is already done by other professionals; then, they will join those meetings. Another remark was that they would only organize or coordinate meetings if the content of those meetings was related to sports and physical activity.

“It is my job if these meetings are related to sports and physical activity. For other content, the coordinating role is played, for example, by the social neighbourhood team. I will participate, but I don’t think it is my job to coordinate those meetings.” (Care sport connector, 142)

## Discussion

This Delphi study was conducted to explore which tasks are beneficial for a care sport connector to facilitate collaboration between the primary care and sports sectors to promote physical activity in relation to other professionals’ perceptions and task profiles. The care sport connectors and other professionals acknowledged that informative and broker tasks are suitable for care sport connectors. However, the other professionals have a wider perception of the care sport connector function than the care sport connectors themselves, and expect that they will fulfil executive and guiding tasks. Regardless of these differences in perception, these results show that there is potential to strengthen the connection between the primary care and sports sectors to promote physical activity. This is especially true because all the professionals acknowledged tasks concerning physical activity promotion and would use the services of a care sport connector. However, the professionals differed about tasks, priorities, the number of tasks and the will to contribute to an intersectoral collaboration.

All professions acknowledged having a task related to physical activity promotion, which is in line with previous studies [23,24,25,26,27,28]. However, in line with our previous interviews and focus groups [10,13], and results of other studies [23,24], general practitioners, nurse practitioners and dieticians mentioned several barriers to fulfilling these tasks (e.g., other priorities, time constraints, a lack of knowledge about sports and physical activities in the neighbourhood and a lack of knowledge about the prescription of physical activity). Care sport connectors and sports club and other physical activity facilities mentioned other barriers (e.g., a difficult target group and too few members to establish profitable groups). Although Dutch policies intend to overcome these, it is not executed in practical local policy. This reflects a gap between the desired and actual promotion of physical activity [23,29] and not aligned subsidies for physical activity promotion [30]. This also relates to the fact that local governments define their own sports and physical activity policies and that they appoint care sport connectors according to their policy goals. Nevertheless, we think these barriers can be overcome through intersectoral collaboration that bundles knowledge, resources and manpower from each profession, with the help of a care sport connector, to expand the reach of interventions and promote physical activity.

However, our study showed that not all professions are willing to collaborate. Although most professions reached consensus about several or all the statements related to collaboration, general practitioners and nurse practitioners did not reach consensus about these statements, which is in line with other studies [23,31]. This is critical because general practitioners and nurse practitioners are at the forefront of primary care, know the target group and their backgrounds, and patients accept their role in discussing lifestyle [32,33,34]. Vision documents about prevention in the general practice setting endorse their important role as a linking pin to other primary care professionals and their pivotal position in strengthening the connection between preventive and curative care [32,35]. In the most recent Dutch competency profile for general practitioners this role is reflected [36]. During the latest Woudschoten conference [37], the term “generalist” has been refined to “medical generalist”. General practitioners indicate that primary prevention is of great importance, with a focus on indicated and care-related prevention for individual patients. The government is particularly responsible for broader, population-oriented prevention tasks and may also involve other healthcare providers, besides general practitioners. A recent national policy is that since 2019 the combined lifestyle intervention for adults with overweight and obesity is reimbursed by the health care insurance [38]. This might help to change general practitioners’ views towards health promotion and to provide care sport connectors the chance to reach and engage general practitioners and lifestyle coaches in implementing combined lifestyle interventions.

However, time for referring adults with overweight and obesity to combined lifestyle interventions and intersectoral collaboration by primary care providers are not reimbursed [34,39,40], where the questioned other professions get a reimbursement for these activities [2,41]. On the other hand, general practitioners and nurse practitioners mentioned that they would collaborate if they found it useful. In our opinion, that reflects the public health mind-set of a profession, which can be ameliorated for general practitioners and nurse practitioners. The shift from care and disease to health and behaviour implies another role for professionals, one in which public health becomes part of a responsible society [42] and a mind-set instead of a point of attention from a specific profession [43].

Our results correspond with the HALL framework factors and clusters [8]. Organizational factors such as different visions and responsibilities and available time complicate intersectoral cooperation. Interpersonal factors, such as attitude towards health promotion and willingness to collaborate seem to be influenced by institutional factors, e.g. policy, and may overrule organizational factors, such as a shared responsibility. This makes it difficult for care sport connectors to fulfil their task to strengthen the connection between primary care, sports and physical activity. Yet, our study revealed that professionals appreciate a care sport connector function and are interested in contacting care sport connectors in their municipality. This is an important addition to previous studies [4,23] that found a health broker role could facilitate intersectoral collaboration. Other professions had a broader view of the care sport connectors’ task profile than the care sport connectors themselves. This broader task profile is in line with the competence profile of care sport connectors [44] and the findings of studies that emphasized the importance of a guiding role and suitable physical activities [45,46] and acknowledgment by care sport connectors [10]. This gives reasons to believe that a care sport connector could facilitate chain-based promotion that uses the strengths and possibilities of each profession, even though not all professions are open to intersectoral collaboration. For example, general practitioners could take the role of authoritative advisor [32,33] and health advocates [35] and nurse practitioners could refer patients to an easily accessible local sports and physical activities, while a care sport connector could guide as a broker and provide information. Other studies [23,24] with general practitioners have found that positive experiences in physical activity promotion lead to the expansion of results and intersectoral collaboration, in which this chain-based promotion could be of major interest. The reimbursement of the combined lifestyle intervention, introduced in 2019 by the health care insurance, is an opportunity for care sport connectors to realise the chain-based promotion [38].

Nevertheless, care sport connectors had a smaller perception of their function regarding an executive and guiding role and we share this opinion for several reasons. On the one hand, we have to consider that – at the moment we conducted this Delphi study – the Ministry of Health, Welfare and Sport appointed only 2,900 Neighbourhood Sports Coaches, of which approximately 7% focus on the connection between primary care, sports and physical activity [47]. The potential target group is huge—50% of people suffer from one or more chronic diseases, and 48% of them do not meet the physical activity Guidelines [48] —and out of balance with the number of available care sport connectors. On the other hand, while the care sport connector function is new, physical activity promotion is not. Each municipality already has some resources, facilities and networks a care sport connector can take advantage of to promote physical activity. Therefore, it makes sense that care sport connectors only reached consensus for their informative and broker roles as a core function, and stick to a more collective execution of their tasks. By covering gaps in resources, networks and facilities, care sport connectors can temporarily fulfil some tasks outside their core function. These tasks can differ due to differences in context and the absence of a blueprint for their function. However, these additional tasks are helpful in facilitating the process of physical activity promotion but should be taken over by other professionals according to the mind-set of public health and the unfeasible demand on care sport connectors. In addition, a collective approach that facilitates the process of collaboration between sectors provides a better perspective for long-term physical activity promotion [42,49,50]. In addition, it should be taken into account that care sport connectors need a broad range of competencies to fulfil all these roles [51].

The national government incorporated these insights in its new policy on neighbourhood sports connectors [52]. The number of care sport connectors has been expanded to 3,625 in 2022 and new goals have been set. The policy focuses on the broker role of the care sport connector and sports for all. Based on our study, our recommendations would be to improve communication between policymakers and care sport connectors, define the role of care sport connectors in facilitating intersectoral collaboration and their needed competences, such as network and communication skills. In addition, it is recommended to support (sustainable) intersectoral collaboration between the primary care and sports sector, e.g. by providing subsidies for intersectoral collaboration and by setting common goals. From a scientific stance, a further recommendation is to facilitate and evaluate intersectoral collaboration continuously, for which the HALL-frameworks [8] offers operationalisation and guidance.

### Strengths and limitations

The strength of this exploratory study is the broad approach used to reveal perceptions from eight professions concerned with physical activity promotion. By using the Delphi method, adapted to suit this particular study [15,17,20], we could determine which tasks and perceptions were acknowledged by a large proportion of the professionals in a profession [15]. Our study resulted in a stable overview of tasks and perceptions across four subsequent rounds and low interquartile ranges within each round. Compared to other Delphi studies, the defined 80% agreement for consensus was quite high [15,18,20,53].

As expected with a Delphi study, participation rates declined each round as effort required by Delphi participants increases [17], but did not decline below the critical number of seven professionals per profession [54], except for Social neighbourhood teams. Therefore, the results for Social neighbourhood teams might be not representative but nevertheless valuable. The social neighbourhood team is a new profession in the Netherlands and this study revealed that social neighbourhood teams want to contribute to physical activity promotion. Study dropout by social neighbourhood teams was mainly because they are in the start-up phase and have no clear idea of their contribution to physical activity promotion.

Another limitation of our study was the recruitment of general practitioners. The response rate after random selection was low, so we approached general practitioners from our own address list to obtain a sufficient number of participants. Despite this bias, we think that our results are representative because they correspond to those of previous studies. However, our results may be a bit optimistic due to professionals’ interests in physical activity promotion. Nevertheless, this still reveals the possibilities and perceptions of the involved professions.

## Conclusion

In conclusion, our results show connecting factors with the potential for care sport connectors to fulfil the expectation to strengthen the connection between primary care, sports and physical activity. All the professionals acknowledged a task concerning physical activity promotion and accepted a broker role, which is the beginning of the public health mind-set. Nevertheless, further development of this mind-set and a collective approach are needed to promote physical activity, particularly in general practice settings.

We must acknowledge several barriers related to policy, which are hard to affect in an executive function such as a care sport connector. Our study found tasks care sport connectors need to perform to overcome other barriers, such as providing information about available physical activities and bringing to light other sites to contact the target group. In addition, we know what we can expect from each profession and how these professions relate to each other. With this information and the overview of expectations, it is possible for care sport connectors to select relevant tasks to strengthen the connection between the primary care and sports sectors to promote physical activity. Chain-based physical activity promotion could be a first step.

Therefore, our results could lead to combining forces to reinforce the public health mind-set even more. The perception of care sport connectors can develop further over time, which is reasonable for a new profession. It would be relevant to investigate how the care sport connector function will develop, which roles are accepted by care sport connectors, if they feel competent, and whether this function will facilitate intersectoral collaboration to promote physical activity in the near future. Thereby, it is important to share this information with care sport connectors and other professionals to make them aware of developments concerning the care sport connector function and this topic. This will make the care sport connector function more transparent and increase familiarity with it.

## Additional Files

The additional files for this article can be found as follows:

10.5334/ijic.4789.s1 Appendix A. Professions’ tasks concerning PA promotion.

10.5334/ijic.4789.s2 Appendix B. Intersectoral collaboration.

10.5334/ijic.4789.s3 Appendix C. Expectations and perceptions of Care Sport Connectors’ tasks.
