# Supplementary material for: Perceptions of Care Sport Connectors’ Tasks for Strengthening the Connection Between Primary Care, Sports and Physical Activity: A Delphi Study
Source: Int J Integr Care. 2020 Apr 1;20(1):13. doi: 10.5334/ijic.4789 (PMC7147677; doi:10.5334/ijic.4789)
Supplement: Appendix A. — Professions’ tasks concerning PA promotion. [file ijic-20-1-4789-s1.pdf]

Appendix A: Professions' tasks concerning PA promotion

**Statements**	**GP**	**NP**	**PH**	**DI**	**SNT**	**MHS**	**SPO**
**Goals:**							
As a professional, it is my task to pay attention to the promotion of a healthy lifestyle	-	-	-	-	-	**0**	-
As a professional, it is my task to pay attention to physical activity promotion in the daily lives of patients	**1***	**1**	**1**	**1**	-	**1**	-
As a professional, it is my task to stimulate residents to be physically active	-	-	-	-	**1**	-	-
**Inform:**	-	-	-	-	-	-	-
As a professional, it is my task to discuss the importance of physical activity with patients	**1**	**1**	-	-	-	-	-
As a professional, it is my task to provide patients insight into the benefits of physical activity	**0**	**1**	**1**	**1**	-	**1**	-
As a professional, it is my task to provide patients insight into the necessity of getting a sufficient amount of physical activity	**0**	**1**	**1**	**1**	-	**1**	-
As a professional, it is my task to provide patients insight into the possibilities for staying physically active in daily life	1	1	**1**	2	**2**	1	-
As a professional, it is my task to stimulate physical activity and sports behaviour through personal contact with the target group	-	-	-	-	0	-	-
As a professional, it is my task to inform the target group about sports and physical activities	-	-	-	-	2	-	-
As a professional, it is my task to enlarge the 'healthy' knowledge of residents	-	-	-	-	-	**1**	-
As a professional, it is my task to accept that a CSC risk cases from a fittest alerts to visit a GP	**0**	-	-	-	-	-	-
As a professional, it is my task to be willing to spread information about sports and physical activities in my practice	**1**	-	-	**1**	-	-	-
As a professional, it is my task to contact the CSC if I identify new questions regarding sports and physical activities	-	1	-	-	**2**	**1**	-
As a sports club, it is our responsibility to notify the CSC about changes in our sports and physical activity offerings	-	-	-	-	-	-	**1**
**Refer:**							
As a professional, it is my task to motivate patients to be physically active in their daily routine	**0**	**1**	**1**	**1**	-	-	-
As a professional, it is my task to effectively provoke patients to become physically active	1	2	-	-	-	-	-
As a professional, it is my task to advise patients about suitable physical activities	-	-	**1**	1	-	-	-
As a professional, it is my task to try to be, as much as possible, aware of the regular sports and physical activities that are present in the neighbourhood	2	0	**1**	**1**	**1**	2	-
As a professional, it is my task to refer patients to a physiotherapist if they are not able to participate in a regular sport or physical activity	**0**	1	-	**1**	-	-	-
As a professional, I will actively refer patients to regular sports and physical activities in the neighbourhood if these are suitable for the patient	2	0	**1**	**2**	-	-	-
As a professional, I will use the social cart to refer people to sports and physical activities	4	**1**	**1**	**1**	**1**	-	-
As a professional, I will put aside my own interests regarding the physical activities in our practice because I think the wish of the patients is outweighed for the choice of a sport or physical activity	-	-	**1**	-	-	-	-
**Execute:**							
As a professional, it is my task to use physical activity as a means	-	-	-	-	4	1	-
As a professional, it is my task to signal needs from the community	-	-	-	-	-	**1**	-
As a professional, it is my task to adapt sports and physical activities to the needs of the target group	-	-	-	-	-	-	2
As a professional, it is my task to ensure suitable companions and trainers for our sport and physical activities offerings	-	-	-	-	-	-	**2***

As a professional, it is my task to be willing to inform trainers and coaches about necessary guidance for people with (a high risk for) health problems	-	-	**1**	**1**	-	-	-
As a sports club, we will welcome people who are referred by a care professional in our sports and physical activity groups	-	-	-	-	-	-	**1**
As a sports club, it is our responsibility to refer people to a care professional if they encounter physical complaints	-	-	-	-	-	-	3
If a CSC asks me to, I am willing to give group sessions to inform people about the need and benefits of sufficient physical activity	4	3	**1**	-	-	-	-
If a CSC asks me to, I am willing to give group sessions to prepare people to participate in regular physical activities	-	-	**1**	-	-	-	-
If a CSC asks me to, I am willing to offer regular physical activities in my practice which are not covered by insurance	-	-	**1**	-	-	-	-
If a CSC asks me to, I am willing to offer sports and physical activities for people with (an increased risk for) health problems	-	-	-	-	-	-	1
If a CSC asks me to, I am willing to be a social involved club	-	-	-	-	-	-	**1**
If a CSC asks me to, I am willing to open our accommodation for neighbourhood activities to realize a neighbourhood function	-	-	-	-	-	-	**1**
If a CSC asks me to, I am willing to permit try-outs for people in the target groups before they decide to become members	-	-	-	-	-	-	**0**
If a CSC asks me to, I am willing to offer flexible memberships	-	-	-	-	-	-	2
If a CSC asks me to, I am willing to arrange starting groups	-	-	-	-	-	-	**1**

Interquartile range is presented for each statement, with a possible range from 0-7; bold number, consensus; bold number*, consensus reached in 4th round due to a lower response rate; other numbers, no consensus reached; - , statement was not provided to this profession.

Abbreviations: GP, general practitioner; NP, nurse practitioner; PH, physiotherapist; DI, dietician; SNT, social neighbourhood team; MHS, municipal health service; SPA, sports and other physical activity facilities; CSC, care sport connector; PA, physical activity
