# Supplementary material for: Perceptions of Care Sport Connectors’ Tasks for Strengthening the Connection Between Primary Care, Sports and Physical Activity: A Delphi Study
Source: Int J Integr Care. 2020 Apr 1;20(1):13. doi: 10.5334/ijic.4789 (PMC7147677; doi:10.5334/ijic.4789)
Supplement: Appendix B. — Intersectoral collaboration. [file ijic-20-1-4789-s2.pdf]

**Appendix B**: Intersectoral collaboration

| Statements | GP | NP | PH | DI | SNT | MHS | SPO |
|---|---|---|---|---|---|---|---|
| **Goal:** | | | | | | | |
| As a professional, I am willing to collaborate with other professionals in the neighbourhood to stimulate residents to be physically active | 2 | 1 | **1** | **1** | **1** | **1** | **1** |
| **Participate in meetings:** | | | | | | | |
| As a professional, I will participate in (network) meetings in the neighbourhood that are dedicated to promoting a healthy lifestyle among neighbourhood residents | 3 | 2 | **1** | **1*** | **1** | **1** | **3*** |
| As a professional, I will participate in (network) meetings in the neighbourhood to become aware of or introduce activities and opportunities that exist in the neighbourhood | 3 | 2 | **1** | 1 | **1** | 1 | **2** |
| As a professional, I will participate in (network) meetings in the neighbourhood to become aware of other health care and sports professionals in the neighbourhood | 3 | 2 | **1** | 1 | **1** | 2 | 1 |
| As a professional, I will participate in (network) meetings in the neighbourhood to discuss how we can work together to stimulate people to become (more) physically active | 3 | 2 | **1** | 2 | - | **1** | **1** |
| **Arranging activities:** | | | | | | | |
| As a professional, I am willing to develop multidisciplinary programs with other professionals to promote the overall lifestyles of participants | 3 | 2 | **1** | 2 | **2** | **1** | 1 |
| As a professional, I am willing to contribute to activities organized in the district to promote a healthy lifestyle (e.g., fitness tests, health fairs) | 3 | 3 | **1** | 1 | **2** | - | **1** |
| As a professional, I am willing to involve residents in organizing sports and physical activities | - | - | - | - | **2** | - | - |
| As a professional, I am willing to develop demand-driven sports or physical activities in collaboration with healthcare providers | - | - | - | - | - | - | 1 |
| **Contact:** | | | | | | | |
| As a professional, I would like to become acquainted with sports and exercise professionals or health and welfare professionals from the neighbourhood | 1 | 1 | **1** | **1** | **1** | 3 | **1*** |
| As a professional, I would like to have contact with sports and exercise groups from the neighbourhood | 2 | 2 | **1** | 2 | **1** | 2 | 2 |
| As a professional, I would like to have contact with professionals from the exercise and sports sector so they can implement exercise and sports interventions | - | - | - | - | - | 1 | - |
| As a professional, I would like to have contact with professionals from the exercise and sports sector so we can reinforce each other | - | - | - | - | - | 2 | - |
| As a professional, I would like to have contact with professionals from the exercise and sports sector so we can utilize each other's expertise | - | - | - | - | - | 2 | - |

Interquartile range is presented for each statement, with a possible range from 0-7; bold number, consensus; bold number*, consensus reached in 4th round due to a lower response rate; other numbers, no consensus reached; - , statement was not provided to this profession.
Abbreviations: GP, general practitioner; NP, nurse practitioner; PH, physiotherapist; DI, dietician; SNT, social neighbourhood team; MHS, municipal health service; SPA, sports and other physical activity facilities; CSC, care sport connector; PA, physical activity
