# Supplementary material for: Perceptions of Care Sport Connectors’ Tasks for Strengthening the Connection Between Primary Care, Sports and Physical Activity: A Delphi Study
Source: Int J Integr Care. 2020 Apr 1;20(1):13. doi: 10.5334/ijic.4789 (PMC7147677; doi:10.5334/ijic.4789)
Supplement: Appendix C. — Expectations and perceptions of Care Sport Connectors’ tasks. [file ijic-20-1-4789-s3.pdf]

Appendix C: Expectations and perceptions of Care Sport Connectors' tasks

**Statements**	**GP**	**NP**	**PH**	**DI**	**SNT**	**MHS**	**SPA**	**CSC**
**Goals:**								
As a professional, I see the work of a CSC as an addition to my work	-	1	**1**	-	-	-	-	-
As a professional, I am open to a CSC contacting me	**0**	1	**1**	**1**	**1**	**0**	**1**	-
As a professional, I do not see the work of a CSC as competition	-	-	**1**	-	-	-	-	-
**Informative:**								
As a professional, I expect a CSC to signal needs from the neighbourhood and share this information	-	-	-	-	-	**1**	-	**1**
As a professional, I expect a CSC to be aware of the sports and physical activities in the neighbourhood	**1**	**1**	**1**	**1**	**1**	**0**	**1**	**1**
As a professional, I expect a CSC to share knowledge acquired from CSCs in other municipalities	-	-	-	-	-	**1**	-	1
As a professional, I expect a CSC to map the sports and physical activities	**1**	**1**	**1**	**1**	**1**	**1**	**2***	**1**
As a professional, I expect a CSC to keep me informed about current sports and physical activities	1	**1**	**1**	**2**	**1**	3	-	**1**
As a professional, I expect a CSC to keep us informed about developments related to sports and physical activities	-	-	-	-	**2**	-	-	**1**
As a professional, I expect a CSC to be aware of opportunities for transferring patients from care and welfare professionals	-	-	-	-	-	-	**0***	**1**
As a professional, I expect a CSC to provide us with information about opportunities for transferring patients from care and welfare professionals	-	-	-	-	-	-	2	**1**
As a professional, I expect a CSC to create awareness of his/her function and its professional potential for us	**1**	**1**	**1**	**1**	**1**	-	-	**1**
As a professional, I expect a CSC to ensure that he/she is known in the neighbourhood among residents and care, welfare and sports professionals	-	-	-	-	-	**1**	-	**1**
As a professional, I expect a CSC to engage citizens in an attractive way for sports and physical activities instead of using an information meeting					**2**	-	-	**0**
As a professional, I expect a CSC to provide insight into the societal role we could play as a club	-	-	-	-	-	-	2	2
As a professional, I expect a CSC to provide an overview of municipal or national (financial) support	-	-	-	-	-	-	1	1
As a professional, I expect a CSC to know about the opportunities and needs of people with (an increased risk for) health problems concerning sports and physical activities	-	-	-	-	-	-	**1***	**2**
As a professional, I expect a CSC to transfers knowledge which is necessary for the provision of sports and physical activities for people with (an increased risk for) health problems	-	-	-	-	-	-	1	**1**
**Executive:**								-
As a professional, I expect a CSC to arrange easily accessible sports and physical activities	**1**	**0**	**1**	**2**	-	**1**	-	**1***
As a professional, I expect a CSC to arrange sports and physical activities that meet the wishes and needs of the target group	-	-	-	-	**2**	-	-	**1**
As a professional, I expect a CSC to organize fitness tests in the municipality	-	-	2	2	-	-	-	1
As a professional, I expect a CSC to provide support to recruit members with (an increased risk for) health problems	-	-	-	-	-	-	**0**	3
As a professional, I expect a CSC to provide support for the guidance of people with (an increased risk for) health problems	-	-	-	-	-	-	1	3
As a professional, I expect a CSC to provide support to align sports and physical activities to the needs and abilities of people with (an increased risk for) health problems	-	-	-	-	-	-	**0***	1
As a professional, I expect a CSC to actively recruit members for sports and physical activities that go beyond our regular offerings	-	-	-	-	-	-	2	2
**Referral:**								
As a professional, I would use the guiding service of a CSC	**0**	**1**	**1**	**1**	**1**	-	-	-
As a professional, I expect a CSC to guide people, when necessary, to suitable sports and physical activities	-	-	-	-	-	**1**	-	1
As a professional, I expect a CSC to develop a buddy system so people can exercise together instead of individually	**1**	**1**	0	**2**	**2**	-	-	2
As a professional, I expect a CSC to align their guidance to the needs and capabilities of a person	**0**	**0**	**1**	**1**	**1**	-	-	1
As a professional, I expect a CSC to arrange demand-driven guidance	-	-	-	-	**1**	-	-	2
As a professional, I expect a CSC to know how to motivate a person to participate in a sport or physical activity	**0**	**0**	**1**	**0**	-	-	-	1

As a professional, I expect a CSC to support people in their choices of suitable sports or physical activities	**0**	-	-	**1**	-	-	-	**1**
As a professional, I expect a CSC to ensure that people make their own decisions regarding a sport or physical activity	-	**0**	-	-	-	-	-	**1**
As a professional, I expect a CSC to direct people to their own responsibility concerning their sport or physical activity behaviour	-	-	-	--	4	-	-	**1**
As a professional, I expect a CSC to monitor people to ensure they have made structural changes in behaviour	2	2	**1**	1	-	-	-	3
As a professional, I expect a CSC to act as a big stick to continue with a physical activity	-	1	-	-	-	-	-	2
As a professional, I expect a CSC to provide feedback about the process	2	2	**1**	2	**1**	-	-	3
As a professional, I expect a CSC to refer people back to me if they have physical complaints	**0**	1	**1**	-	-	-	-	**1**
As a professional, I expect a CSC to refer people back to me if they quit or do not show up at sports or physical activities	4	3	**1**	-	-	-	-	3
As a professional, I expect a CSC to guide people towards regular sports or physical activities after their work with a physiotherapist	-	-	4	-	-	-	-	1
As a professional, I expect a CSC to align with a physiotherapist if a person is ready to be physically active in regular activities and my role as a CSC can be fulfilled	-	-	**1**	-	-	-	-	**1**
As a professional, I expect a CSC to also guide people to physical activities in our practice	-	-	**1**	-	-	-	-	2
As a professional, I expect a CSC to also refer participants from other physical activities to our practice when they suffer from physical complaints	-	-	**1**	-	-	-	-	1
As a professional, I expect a CSC to guide people simultaneously to their therapy in my practice	-	-	-	1	-	-	-	4
As a professional, I expect a CSC to accompany us by visiting a person to guide him/her to a suitable sport or physical activity	-	-	-	-	3	-	-	2
**Broker role:**								
As a professional, I expect a CSC to be flexible regarding working hours	2	-	-	-	-	-	-	**1**
As a professional, I expect a CSC to take a coordinating role concerning (network) meetings in the municipality	**0**	**0**	**1**	**1**	2	1	**1***	1
As a professional, I expect a CSC to take a coordinating role to connect care, welfare and sports professionals in the neighbourhood	-	-	-	-	-	**1**	-	**1**
As a professional, I expect a CSC to establish new connections between the first and zero lines in the community to expand the network	-	-	-	-	-	1	-	**1**
As a professional, I expect a CSC to organize meetings for care, welfare and sports professionals on a structural basis	-	-	-	-	-	1	-	2
As a professional, I expect a CSC to……proceed with a neighbourhood-oriented approach	-	-	-	-	-	**1**	-	3
As a professional, I expect a CSC to set up a sports platform in the community	-	-	**0**	-	-	-	-	1
As a professional, I expect a CSC to also focus on general health, instead of only promoting physical activity	-	-	-	-	-	**1**	-	-
As a professional, I prefer the CSC to act as an intermediary for the contact with sports and PA facilities or care professionals instead of maintaining contact with these professionals myself	**1**	0	**0***	**1**	4	**1**	2	**1**
As a professional, I am open to a CSC taking a position in (the outer layer of) the social neighbourhood team	-	-	-	-	**1**	-	-	-
If a CSC asks me to, I am willing to help the CSC write project proposals to promote physical activity	-	-	-	-	-	**1**	-	-
If a CSC asks me to, I am willing to help the CSC draft a plan of action for a physical activity intervention	-	-	-	-	-	**0**	-	-
If a CSC asks me to, I am willing to introduce a CSC to our network of healthcare professionals	-	-	-	-	-	**1**	-	-
If a CSC asks me to, I am willing to help the CSC bring together professionals from the care, welfare and sports sectors	-	-	-	-	-	**1**	-	-
If a CSC asks me to, I am willing to organize information sessions about healthy lifestyles	-	-	-	-	-	1	-	-
If a CSC asks me to, I am willing to share the identified community needs	-	-	-	-	-	**1**	-	-
If a CSC asks me to, I am willing to share the knowledge we gain from research	-	-	-	-	-	**1**	-	-

Interquartile range is presented for each statement, with a possible range from 0-7; bold number, consensus; bold number*, consensus reached in 4th round due to a lower response rate; other numbers, no consensus reached; - , statement was not provided to this profession.

Abbreviations: GP, general practitioner; NP, nurse practitioner; PH, physiotherapist; DI, dietician; SNT, social neighbourhood team; MHS, municipal health service; SPA, sports and other physical activity facilities; CSC, care sport connector; PA, physical activity
